# Neonatal familial Evans syndrome associated with joint hypermobility and mitral valve regurgitation in three siblings in a Saudi Arab family

**DOI:** 10.4103/0256-4947.51784

**Published:** 2009

**Authors:** Fathelrahman E. Ahmed, Mohameed S. AlBakrah

**Affiliations:** From the Department of Pediatrics, North West Armed Forces Hospital, Tabuk, Saudi Arabia

## Abstract

The occurrence of autoimmune hemolytic anemia and immune thrombocytopenia in the absence of a known underlying cause led to the diagnosis of Evans syndrome in a 9-month-old male. Subsequently, a similar diagnosis was made in two siblings (a 3-year-old boy and a 1-day-old girl). The 9-month-old had a chronic course with exacerbations. He was treated with steroids, intravenous immunoglobulin and colchiccine with a variable response. He died of congestive heart failure at the age of 8 years. The brother's disease course was one of remission and exacerbation. With time, remissions were prolonged and paralleled an improvement in joint hypermobility. The sister died of sepsis after a chronic course with severe exacerbattions. Only two families with Evans syndrome have been reported in the English medical literature. In one report (in a Saudi Arab family), the disease was associated with hereditary spastic paraplegia.

Evans syndrome is the occurrence of a Coombs positive hemolytic anemia and immune thrombocytopenia without a known underlying etiology.^1^ It is a chronic immunological disorder with a variable course, and the exact pathophysiology is unknown.^2^ The disease can present in the neonatal period.^3^ There are rare reports of familial Evans syndrome.^4^,^5^ Joint hypermobility, not a disease in itself, is defined as an abnormally increased range of joint motion due to excessive laxity of the constraining soft tissues. Hypermobility is determined according to the criteria of Beighton et al.^6^ Patients are given a score of 0-9, one point being allocated for the ability to perform each of the tests: 1) passive dorsiflexion of the little finger beyond 90^°^; 2) passive apposition of the thumb to the flexor aspects of the forearm; 3) hyperextension of the elbow beyond 10°; 4) hyperextension of the knee beyond 10°; and 5) forward flexion of the trunk, with the knees straight, so the palms of the hands rest easily on the floor. Patients are considered hypermobile if they score 4 or more out of 9.

Hypermobility may be a benign syndrome or part of a genetic disorder like Ehlers-Danlos syndrome or Marfan syndrome. All have strong genetic components. 7–9 The benign type is commonly inherited as an autosomal dominant, although autosomal recessive and X-linked transmission also have been documented.^9^ We describe three siblings with Evans syndrome, generalized joint hypermobility, and mitral valve prolapse causing mitral regurgitation. The parents of these children are first cousins and have two other children. There was no anemia, thrombocytopenia or joint hypermobility in these other children or their parents. The father is married to another wife, a first cousin as well, with five children. The children and their mother are free of anemia, thrombocytopenia and joint hypermobility.

## CASE 1

A full-term Saudi male infant, a product of a first cousin consanguineous marriage, developed jaundice in the first day of life. The mother's blood group was O and Rh negative. The infant's blood group was A positive, and the father's blood group was A positive. Serum bilirubin was 231 μmol/L (reference range, 5-17 μmol/L). Hemoglobin was 130 g/L (reference range, 145-185 g/L), the platelet count was 91×10^9^/L (reference range, 150-450×10^9^/L) and the direct agglutination test (DAT) was strongly positive. An extended blood group (Kell, Duffy and Kid) showed no minor group incompatibility. He was diagnosed with ABO incompatibility and treated with phototherapy for two days and discharged. He presented in January 1991, at the age of 9 months, with recurrent epistaxis and petechiae of 3 months' duration. He was pale, jaundiced, with multiple petechiae and generalized joint hypermobility (Beighton score of 8/9). There was clinical evidence of mitral regurgitation. An echocardiogram revealed mitral valve prolapse (MVP) and severe mitral regurgitation. There were no features of known collagen disease or syndromes involving joint hypermobility. Laboratory tests showed a hemoglobin of 51 g/L (reference range, 114-127 g/L), WBC of 3.3×10^9^/L (reference range, 6-17.5×10^9^/L), a platelet count of 9×10^9^/L (reference range, 150-450×10^9^/L), a reticulocyte count of 7.6% (reference range, 0-1%) and a positive Coombs test. The bone marrow was hypercellular with an increased number of megakaryocytes. Immune-phenotyping and serum immunoglobulin levels were normal and antinuclear antibodies (ANA) were negative. Antiplatelet antibodies were not obtained. Evans syndrome was diagnosed. He was treated with steroids, intravenous immunoglobulin and colchicine with a variable response. He ran a chronic course with exacerbations. Episodes of epistaxis and petechiae were observed in the presence of a platelet count of 80 to 90×10^9^/L with no response to different modalities of treatment. He died, at the age of 8 years, of congestive heart failure secondary to severe mitral regurgitation following chordal rupture, which was confirmed by an echocardiogram while awaiting acceptance to a cardiac center.

## CASE 2

A brother of the first patient, this male infant, born at term, had jaundice on the first day of life. Laboratory data showed a serum bilirubin of 211 μmol/L (reference range, 5-17 μmol/L), a hemoglobin of 175 g/L (reference range, 145-185 g/L), white blood count of 9.7×10^9^/L (reference range, 9.4-34×10^9^/L), a platelet count of 114×10^9^/L (reference range,150-450×10^9^/L), nucleated red blood cells 14/100 white blood cells (reference range, 0-5 nucleated RBC/100 WBC) and strongly positive DAT. The patient's blood group was O positive and the mother's blood group was O and Rh negative. An extended blood group was not obtained. He was diagnosed as having minor group incompatibility, treated with phototherapy for one day, and discharged. He was lost to follow-up until October 1995 (age 3 years), when he presented with epistaxis and petechiae. He was pale, with petechiae and generalized joint hypermobility (Beighton score 8/9). There was cardiomegaly with a mitral regurgitation murmur. An echocardiogram showed mitral valve prolapse and moderate mitral regurgitation. There were no features indicative of known collagen disorders or other syndromes involving joint hypermobility.

The laboratory data showed WBC of 8.1×10^9^/L (reference range, 5.5-15.5×10^9^/L), hemoglobin of 83 g/L (reference range, 115-125g/L), a platelet count of 7.0×10^9^/L (reference range, 150-450×10^9^/L), a reticulocyte count of 6.3% (reference range, 0-2%) and strongly positive DAT. The bone marrow examination revealed many megakaryocytes. Leukocyte phenotype by flow cytometry, cytogenetic studies, immunoglobulin levels and thyroid function were normal. ANA and rheumatoid factor (Rh factor) were negative. DNA analysis was not obtained due to parent refusal. An extended blood group (Kell, Duffy and Kid) showed no minor group incompatibility. Evans syndrome was diagnosed based on the presence of Coombs positive hemolytic anemia and thrombocytopenia. He experienced recurrent and prolonged attacks of epistaxis with a platelet count between 70 and 90×10^9^/L. Coagulation screening was normal apart from a prolonged bleeding time (12 minutes) (reference range, 2-7 minutes). He showed variable responses to treatment with steroids and intravenous immunoglobulin. His clinical course was that of remissions and exacerbations. At the time of writing he was in clinical remission for 3 years without treatment. His platelet count was 70-80×10^9^/L and he had no further episodes of epistaxis. His joint hypermobility improved (Beighton score=4/9). The mitral valve regurgitation murmur disappeared clinically, but a mild regurgitation was found on an echocardiogram. This coincided with improvement in Beighton score and bleeding episodes.

## CASE 3

The 1-day-old sister of the first two cases had jaundice when first seen in October 2002. Clinical examination showed generalized joint hypermobility (Beighton score 8/9) and mitral regurgitation murmur. Laboratory data showed a hemoglobin of 131 g/L (reference range, 145-185 g/L), a leukocyte count of 7.3×10^9^/L (reference range, 9.4-34.0×10^9^/L), a platelet count of 91×10^9^/L (reference range, 150-450×10^9^/L) and a normal reticulocyte count. The infant's blood group was A positive with a positive DAT. An extended blood group (Kell, Duffy and Kid) showed no minor group incompatibility. She was diagnosed as having Evans syndrome, treated with phototherapy for 3 days, and discharged. At 18 months of age, she presented with epistaxis. She was clinically pale, jaundiced, with generalized petechiae. The Beighton joint score was unchanged (8/9). Mitral valve prolapse with moderate regurgitation was diagnosed and confirmed by an echocardiogram. There were no symptoms or signs indicating known collagen disorder or other syndromes involving joint hypermobility. Her laboratory data showed a hemoglobin of 71 g/L (reference range, 105-125 g/L), leukocyte count of 3.8×10^9^/L (reference range, 6.0-17.0×10^9^/L), a platelet count of 7.1×10^9^/L (reference range, 150-450×10^9^/L), total serum bilirubin 131 μmol/L (reference range, 5-17 μmol/L) and the reticulocyte count was 7.4% (reference range, 0-2%). DAT was positive. Serum immunoglobulin level and leukocyte phenotype by flow cytometry were normal. An HIV test and ANA were negative, and a coagulation screen was normal except the bleeding time, which was prolonged (11.5 minutes) (reference range, 2-7 minutes). The bleeding time remained prolonged even when the platelet count was more than 100×10^9^/L. DNA analysis was not obtained due to parent refusal. She responded initially to intravenous methylprednisolone and intravenous immunoglobulin, but then ran a chronic course with exacerbation. She had a mitral valve repair, for congestive cardiac failure, with no significant bleeding. Postoperatively, she entered a chronic severe intravascular hemolytic course, which was refractory to different modalities of treatment including cyclosporine, intermediate doses of cyclophosphamide and infliximab. She died of Pseudomonas septicemia at 33 months of age.

## DISCUSSION

Neonatal Evans syndrome, reported once in the literature,^3^ developed in three consecutive siblings over an 11-year period. All had jaundice, thrombocytopenia and a positive DAT on the first day of life. In the first patient, neonatal Evans syndrome was not considered because he was thought to have a combination of an ABO incompatibility (the mother's blood group was “O” and the baby's blood group was “A”) and rhesus isoimmunization (the mother was Rh negative and the baby was Rh positive). In retrospect, the absence of Rh antibodies in the mother excluded Rh incompatibility, and the presence of thrombocytopenia and strongly positive DAT favors Evans syndrome rather than ABO incompatibility. In the second patient, minor group incompatibility was a diagnosis of exclusion, as an extended blood group was not obtained in the neonatal period. However, the positive DAT at the age of three years can not be due to minor group incompatibility even if it was present. The normal serum immunoglobulin and the negative ANA made immune deficiency and SLE, respectively, unlikely. In the third patient, the diagnosis of Evans syndrome was made easy by the family history.

There are two reports of familial Evans syndrome in the English literature. McLeod et al reported Evans syndrome in three siblings who had no other inherited disorder.^5^ We reported two brothers, a product of a first-cousin marriage, with Evans syndrome and an autosomal recessive hereditary spastic paraplegia.^4^ The addition of this family further supports a genetic predisposition, most likely an autosomal recessive inheritance pattern, in some cases of Evans syndrome.

The clinical course of multiple recurrences and the use of multiple treatment modalities to control the disease are consistent with reported results by Matthew et al.^10^ However, our patients manifested attacks of epistaxis and petechiae out of proportion to their platelet count. Mild bleeding associated with an increased capillary fragility was reported in several genetic collagen disorders.^11^ Prolonged bleeding has also been reported in patients with hyperflexible thumbs (one of the diagnostic criteria of joint hypermobility).^12^ The latter might explain the prolonged bleeding episodes and bleeding time despite near normal platelets count observed in the third case. The lack of significant bleeding during and after cardiac surgery does not exclude this possibility, as a prolonged bleeding time does not predict a bleeding risk.^13^ This is important to realize when Evans syndrome occurs in patients with joint hypermobility, otherwise, the bleeding tendency might be attributed to thrombocytopenia while it is not. This recognition might save the patient from being subject to further treatment.

Generalized joint hypermobility, measured according to Beighton criteria, was present in all patients. The lack of features suggestive of the serious genetic syndrome, and the significant reduction that occurred in Beighton score in the second case as he grew older suggests the benign form of joint hypermobility. The presence of joint hypermobility in the third case, a girl, excludes an X-linked transmission. The lack of joint hypermobility in the parents, who are first cousins, favors an autosomal recessive mode of inheritance in this family.

Deficiency and haploinsuffiency of tenascin-X, a large extracellular glycoprotein, has been identified as a cause of joint hypermobility in patients with benign joint hypermobility syndrome and the mobility type-Ehlers-Danlos syndrome.^14^ Tenascin-X forms a genetic unit with CYP21B, C4A, and C4B, termed the RCCX module. This region is highly susceptible to recombination events leading to diverse diseases; for example, systemic lupus erythematosus (SLE) is a consequence of C4A deficiency. IgA deficiency is caused by C4B mutation and a deficiency of CYP21B results in congenital adrenal hyperplasia.^14^ The development of autoimmune hemolytic anemia and autoimmune thrombocytopenia is not infrequent in patients with selective IgA defiency 15–17 and SLE.^18^ Adult patients with SLE were more hypermobile (48%) than a control group (15%).^19^ This suggests a pathogenetic relationship between Evans syndrome, not infrequently present with SLE, and joint hypermobility.

In a recent report from Turkey the incidence of joint hypermobility, as judged by Beighton score, was found to be higher in patients with mitral valve prolapse than controls.^20^ Our three patients manifested joint hypermobility with mitral valve prolapse and regurgitation.

The expression of mitral valve prolapse is variable within families.^21^ Weakness of the valvular and restraining chordal tissue with sudden chordal rupture causing abrupt clinical deterioration is a recognized feature.^21^ This was the cause of death in the first patient. Progressive mitral regurgitation causing heart failure, another feature of mitral valve prolapse,^21^ developed in the third patient and was treated surgically. The second patient showed an improvement with time, another feature of mitral valve prolapse.^21^

In conclusion, familial Evans syndrome, possibly an autosomal recessive disorder, may be mistaken for alloimmune hemolytic anemia when it presents in the neonatal period. Although the association of Evans syndrome with joint hypermobility (a genetic collagen disorder) may be a coincidence, a pathogenetic relationship needs to be considered.
